# Practical Medicine, &c.

**Published:** 1846-01

**Authors:** 


### PRACTICAL MEDICINE, &c.

We extract the following conclusions in regard to the comparative merits of the Quinine and Salicine, from a report in the New Orleans Medical and Surgical Journal for Nov. 1845, of a trial in the wards of the Charity Hospital of that city, under the care of Dr. Fenner. The trials were made in the same manner as those dictated by the Surgeon General in the Medical Department of the U. S. Army.

* * * Thus have I given careful observations, of the effects of salicine in twenty cases of intermittent fever, taken promiscuously, and all occurring within a short period. I had commenced its use in two other cases, and would willingly have reported a much larger number, but desisted on account of *the expense* of the remedy. Perhaps no place in the world presents greater advantages for observations upon this disease, than the N. O. Charity Hospital. It is very tedious either to prepare or examine reports of this kind; but if faithfully executed, they offer the best medium of information, both in regard to the effects of remedies, and the nature and progress of disease. It is hoped that these twenty cases will prove in some degree instructive. Let us sum up their results and see what conclusions they will authorize. Although the statistics may not be mathematically exact, they are sufficiently so for our purpose.

Total amount of medicine given in the 20 cases: salicine ℥ vii, ʒ vi, ℈ ii; piperine, ʒ i, ℈ii; quinine ʒ iv.

Largest amount given in any case: salicine, ʒ vii; piperine, ʒ iss; quinine, ʒ iii, ℈ i.

Smallest amount given in any case; salicine, ʒ i, grs x; piperine, grs xii; quinine, ℈ ii.

Average amount of salicine to each patient ʒ iii, grs viii.

It was first tried alone, in all the 20 cases.

It succeeded when given alone, in 11 cases.

It failed when given alone, in 9 cases.

Of these, it succeeded when combined with piperine, in 4 cases.

Quinine had to be resorted to in 6 cases.

Average time of sickness previous to treatment, 7½ days.

Do " " under treatment 6½ days.

Greatest number of parox., after sal. was commenced, 6 days.
Smallest " " " " " 0 "
Average " " " " " 9 "

There were one or more paroxysms after salicine was commenced, in 14 cases.

There were none in 6 cases.

I am informed by the Apothecary of the Hospital that the cost of salicine was $2 00 per oz. Consequently, the value of the amount used in these 20 cases, (say 7¾ oz.,) was about $15 50; and of the average amount given to each patient, (say ʒ iii,) 75 cents. The article appeared to be fresh and genuine.

I prescribed it in the forms of solution, powder and pill; in doses varying from *five grains to one drachm*, and at intervals of from one hour to twelve.

The general effects of the remedy appeared to be *tonic and diaphoretic*. The appetite and strength were generally improved, and the sweating was profuse. I observed no unpleasant effect that I could attribute to the remedy. Where it failed *to do no good it did no harm*.

### *Comparative Statement of 20 Cases of Intermittent Fever treated with the Sulphate of Quinine.*

After the foregoing observations on the use of salicine were completed, I resolved to note 20 cases of intermittent fever treated mainly with quinine; with the view of ascertaining the relative *efficacy* and *cost* of the two remedies. Twenty recent admissions were taken throughout the wards of the Hospital, and of course under the care of different physicians. Upon inquiry I found that no two of them administered the same remedy alike; some of them prescribe it in large doses, and at long intervals; others, the reverse; some give it alone; others, in combination with blue mass, opium, or morphia. As minute notes were taken of these 20 cases, as of the preceding; but for fear of wearying the reader, I will only give the *results*, and the conclusions to which they brought me.

Whole amount of quinine used in the 20 cases, about ℥ iss.
Largest amount given in any case, ʒ i.
Smallest amount given in any one case, grs. xviii.
Average amount of quinine given to each patient, grs. xxxvi.
It was given combined with sulph. morph. grs. xii, to gr. ½, in 2 cases.
" " with ext. opii, grs. xviii, to gr. i, in 4 cases.
" " with blue mass; grs. vi, to grs. x, in 1 case.
" " alone in 13 cases.

*All the cases were promptly cured.*

Average time of sickness before admission; 10 days.
" " " after " 4 "
Greatest number of parox., in any case after taking qui., 2 days.
Smallest " " " " " 0 "
Average number of paroxysms 3[5 "

There were one or more paroxysms after taking quinine, in 10 cases.

There were none, in 10 cases.

The cost of quinine was $3 25 pr. oz. consequently the value of the whole amount used in these 20 cases, (say ½ oz.) was $4 87½, and the average amount (36grs.) about 25 cts.

Candour compels me to state that the cases treated by salicine were generally more severe, than those treated by quinine; it will be recollected that one of them was so malignant as to be with difficulty saved by upwards of ʒ iii of quinine, after having previously taken ʒ iiiss of salicine. The salicine cases occurred chiefly in the months of June and July, when intermittents usually assume their worst forms; the quinine cases all occurred about the first of October, when the disease is generally mildest. These circumstances are worthy of grave consideration, lest we be induced to underrate the actual virtues of salicine. However, taking the two sets of cases as they are presented to us in the foregoing comparative statement, and reviewing the effects of the two remedies in their various combinations before mentioned, we are brought to the conclusion that *the average amount of quinine required to cure 20 cases of intermittent fever, and costing 25 cents, is fully three times as efficacious as the average amount of salicine required in a like number of cases, and costing 75 cents.*

The comparison made in this instance cannot however be considered a perfectly fair one; but when the foregoing reports are taken in conjunction with others that will doubtless be made from the medical department of the Army, they may aid in leading us to a proper estimate of the virtues of salicine.

How many of the foregoing cases would have had their paroxysms broken up merely by *the change of residence, and attention to regimen, without any medicine whatever*, must remain a matter of conjecture. My own opinion is, there would have been a goodly number. Hence the importance of exercising a sound judgment and careful observation in regard to the action and comparative value of medicines. E. D. F.

Oct. 17, 1845.

